# Disorders of MicroRNAs in Peripheral Blood Mononuclear Cells: As Novel Biomarkers of Ankylosing Spondylitis and Provocative Therapeutic Targets

**DOI:** 10.1155/2015/504208

**Published:** 2015-07-26

**Authors:** Qing Lv, Qiuxia Li, Peizhuo Zhang, Yingjuan Jiang, Xinwei Wang, Qiujing Wei, Shuangyan Cao, Zetao Liao, Zhiming Lin, Yunfeng Pan, Jianlin Huang, Tianwang Li, Ou Jin, Yuqiong Wu, Jieruo Gu

**Affiliations:** ^1^Department of Rheumatology, The Third Affiliated Hospital of Sun Yat-Sen University, 600 Tianhe Road, Guangzhou 510630, China; ^2^Shanghai GenePharma Co., Ltd, Shanghai 200000, China

## Abstract

*Background*. MicroRNAs can potentially regulate every aspect of cellular activity. In this study, we investigated whether AS pathogenesis involves microRNAs disorders. *Result*. The expression of 2 microRNAs, hsa-miR-126-3p and hsa-miR-29a, was significantly lower in active AS group before etanercept therapy than in control group. Marched fold changes of them were 3.76 and 16.22. Moreover, expressions of hsa-miR-126-3p and hsa-miR-29a were dramatically upregulated after 12-weeks etanercept treatment. Fold changes were 2.20 and 3.18. All regulations of microRNAs expression mentioned before were statistically significant (fold change >2 and *P* < 0.05). The expression disorders of the 2 microRNAs did not statistically significantly correlated with BASDAI, CRP, and ESR. *Conclusion*. AS pathogenesis involved dysregulation of microRNAs. Hsa-miR-126-3p and hsa-miR-29a will probably become the potential biomarkers and provocative therapeutic targets of AS.

## 1. Background

Ankylosing spondylitis (AS) is a member of a group of rheumatic diseases that affects the axial joints (spine and pelvis), collectively known as spondyloarthropathies. It is a common disease affecting approximately 0.5% of white Europeans and has a global distribution with the exception [1]. The risk of developing the disease is largely genetically determined, and the genetic susceptibility to AS is confirmed by twin study [2]. HLA-B27 is one of the convincing genetic factors [3–5], but it can explain no more than 30% of the overall genetic risks of AS [6]. Much of AS related genetic disorder, outside HLA-B27, still remains to be explored.

MicroRNAs are endogenous ~22 nt RNAs that comprise one of the more abundant classes of gene regulatory molecules in multicellular organisms and likely influence the output of many protein-coding genes [7]. MicroRNAs take part in regulation of gene expression; moreover about one-third of all mRNAs may be regulated by microRNAs [8]. It is now clear that microRNAs can potentially regulate every aspect of cellular activity, including differentiation and development, metabolism, proliferation, apoptotic cell death, viral infection, and tumorigenesis [9].

It was validated that microRNA disorder was related to pathogenesis of rheumatism. Expression of miR-132 and 3 other microRNAs differentiated patients with RA or OA from HC [10], while miR-146a was thought to be biomarker of SLE [11]. The paper also shows that microRNAs may have correlation with pathological changes of AS [12]. Huang et al. found that patients with AS compared to controls had significantly higher levels of miR-21, PDCD4 mRNA, and CTX [13]. To validate whether microRNAs could be biomarkers and therapeutic targets of AS, we investigated the correlation of peripheral blood mononuclear cells (PBMCs) microRNA disorders and AS activity and measured the regulation of microRNAs expression after etanercept therapy.

## 2. Methods

### 2.1. Patients and Control Samples

Forty patients who fulfilled the Modified New York Criteria for Ankylosing Spondylitis (1984) were included in this study. It was demanded that the Bath Ankylosing Spondylitis Disease Activity Index (BASDAI) [14] and VAS of all patients in this study be at least 4. All of them accepted regular etanercept therapy (50 mg, qw, hypodermic injection) for 12 weeks. Immunosuppressive drugs and other medication were not allowed during etanercept therapy. At least 10 mL peripheral blood was obtained from AS patients at baseline and after 12-week therapy. And their clinical information, including BASDAI, CRP, and ESR, was also collected. Fifty healthy volunteers with age and sex matched were taken as controls (Figure 3). Blood samples were collected with ethylenediaminetetraacetic acid (EDTA) containing tube to separate plasma and were stored in 4°C refrigerator. Ethical approval for this study was granted by the Ethics Committee of Third Affiliated Hospital of Sun Yat-sen University. Written permission was obtained from all subjects who participated in the study.

### 2.2. Filtrated MicroRNA by Microarray

PBMCs of 35 AS patients and 47 controls were extracted by using lymphocytes separation medium. Total RNA and microRNAs of each sample were isolated according to manufacturer's protocol of TRIzol Reagent (Life Technologies, Inc.) and mirVana microRNA Isolation Kit (Ambion, Inc.). To confirm that the report of microarray is reliable, the OD value (DU520 UV/Vis Spectrophotometer, Beckman Coulter, Ltd) and electrophoresis (Bio-Rad Mini-Sub GT System, Bio-Rad, Ltd) were used to evaluate the quantity and quality of total RNA. Then microRNAs were marked with Monoreactive Cy3 dye (Amersham Pharmacia Biotech, Ltd) and purified according to mirVana microRNA Labeling Kit (Ambion, Inc.) procedure. Prepared microRNAs were hybridized to microarray probes. 428 microRNAs probes were involved, and all of them were 34–44 nt and had the same Tm value. Fluorescent signals were scanned (Generation iii array scanner, Amersham Pharmacia) and translated into digital signals (Imagequant 5.0, Array Vision 6.0). Relative expression levels of target microRNAs were estimated according to digitized intensity of fluorescence. Calibrator was the median of all the valid data. Average microRNA expression level of each group and the ratio of any two of the three groups (AS patients before treatment, AS patients after treatment, and healthy donors) were independently calculated. Because only CY3 was used to mark target microRNA, the ratio >3 or <0.33 was thought to be statistically significant.

### 2.3. Reverse Transcription and Quantitation of miRNAs by Real-Time PCR

Thirteen microRNAs were chosen for validation, as follows. The relative expression levels of target microRNAs fulfilled being (a) significantly different between AS patients and healthy donors, (b) increasing/decreasing dramatically after regular etanercept therapy, or (c) taking part in the process of inflammation or bone metabolism according to the papers published before. Reverse transcription was performed according to the protocol. Real-time polymerase chain reaction (PCR) was performed on MX-3000P Real-Time PCR Instrument (Stratagen, US) using Beacon Real-Time PCR Universal Reagent (Cat# GMRS-001, GenePharma, Shanghai) and with U6 snRNA as the internal control. Primers were designed as follows. 10 samples of AS patient and 10 control samples were included in pilot real-time PCR experiment. Two microRNAs, hsa-miR-29a and hsa-miR-126-3p, were involved in next step real-time PCR validation. Sample size of each group was enlarged in further study. Relative copy numbers of target microRNAs were obtained. The expression levels of target microRNAs in each sample were calculated according to the copy numbers of target microRNAs. A fold change of >2 was considered significant.

Primers were designed as follows: hsa-miR-let7a (F primer: GGACTGAGGTAGTAGGTT, R primer: CATCAGATGCGTTGCGTA), hsa-miR-let7f (F primer: GGACTGAGGTAGTAGATTG, R primer: CATCAGATGCGTTGCGTA), hsa-miR-let7i (F primer: GGACCTGCGCAAGCTAC, R primer: CATCAGATGCGTTGGCTA), hsa-miR-21 (F primer: GGACTAGCTTATCAGACTG, R primer: CATCAGATGCGTTGCGTA), hsa-miR-26b (F primer: GGACTTCAAGTAATTCAGGA, R primer: CATCAGATGCGTTGCGTA), hsa-miR-27a (F primer: GGACTTCACAGTGGCTAA, R primer: CATCAGATGCGTTGCGTA), hsa-miR-29a (F primer: GGACTAGCACCATCTGAA, R primer: CATCAGATGCGTTGCGTA), hsa-miR-29b (F primer: GGACTAGCACCATTTGAAA, R primer: CATCAGATGCGTTGCGTA), hsa-miR-98 (F primer GGACTGAGGTAGTAAGTTG, R primer: CATCAGATGCGTTGCGTA), hsa-miR-202 (F primer: GGACTTCCTATGCATATAC, R primer: CATCAGATGCGTTGCGTA), hsa-miR-494 (F primer: GGACTGAAACATACACGG, R primer: CATCAGATGCGTTGCGTA), hsa-miR-526a (F primer: GGACCTCTAGAGGGAAG, R primer: CATCAGATGCGTTGCGTA), hsa-miR-126-3p (F primer: GGACTCGTACCGTGAGTA,R primer: CATCAGATGCGTTGCGTA).

### 2.4. Statistical Analysis

Data were presented as the mean ± standard deviation. Statistical analyses were performed using SPSS 10.0. Differences between two groups were analyzed with Wilcoxon rank sum test. Correlations of clinical presentations and microRNA expression levels were also analyzed. Spearman correlation coefficients were calculated. A *P* value less than 0.05 was considered statistically significant.

## 3. Results

### 3.1. Patients and Control Samples

There were 40 AS patients and 50 healthy volunteers included in this study. The male to female ratio and average age in the AS patients group matched with the healthy control group. Means of disease duration, BASDAI, BASFI, CRP, ESR, and medications used were calculated (Table 1).

### 3.2. The Result of Microarray

**Figure 1:**
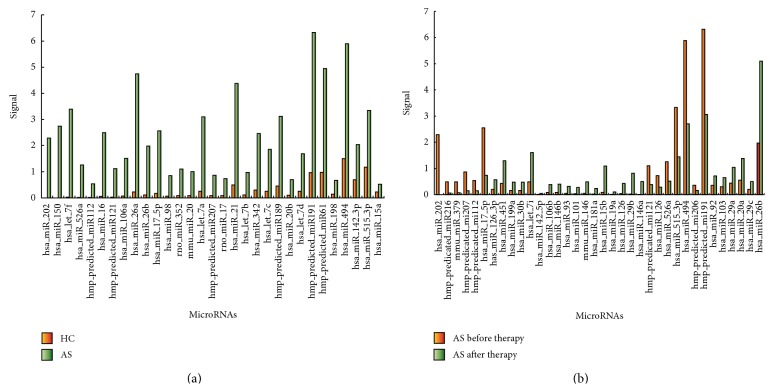
Calibrated fluorescence intensity of microarray in different groups. (a) The microRNAs signal intensity of AS group was compared with that of healthy control. There were 31 microRNAs in this figure. 26 of them, at the left side of the figure, expressed significantly higher in AS group than in HC. The other 5, at the right side of the figure, were probably higher in AS group than in control group. (b) The microRNAs signal intensity of AS group before and after etanercept therapy was compared. There were 36 microRNAs in this figure. 23 of them, at the left side of the figure, had definite expression regulation after therapy. Among them, the expression levels of 6 downregulated significantly after regular etanercept therapy, while 17 upregulated. However, the other 13 microRNAs, at the right side of the figure, were thought to keep different expression levels, not so dramatically changed in AS group before and after etanercept therapy. 7 among them downregulated, while 6 upregulated. AS, ankylosing spondylitis; HC, healthy control.

According to the microarray, there were 26 significantly differentially expressed microRNAs. The expression levels of all the microRNAs were significantly higher in AS group than in control group (fluorescence intensity ratio of AS group to control group was >3 : 1) (Figure 1(a)). Furthermore, the first screening with microRNAs discovered 23 microRNAs, expressions of which were significantly different before and after 12-week etanercept therapy. Among them, the expression levels of 6 microRNAs downregulated significantly after regular etanercept therapy, while the other 17 microRNAs upregulated (fluorescence intensity ratio of AS group after treatment to before treatment was <1 : 3 or >3 : 1) (Figure 1(b)).

However, hsa-miR-15a, hsa-miR-515-3p, hsa-miR-198, hsa-miR-494, and hsa-miR-142-3p were probably higher in AS group than in control group (fluorescence intensity ratio of AS group to control group was >2 : 1 but <3 : 1) (Figure 1(a)). Similarly, 13 microRNAs were thought to keep different expression levels in AS group before and after etanercept therapy. The expression levels of 7 among them probably downregulated after regular etanercept therapy (fluorescence intensity ratio of AS group after treatment to before treatment was <0.5 but >0.3). And the levels of the other 6 probably upregulated after therapy (fluorescence intensity ratio of AS group after treatment to before treatment was <1 : 2 but >1 : 3) (Figure 1(b)).

**Figure 2:**
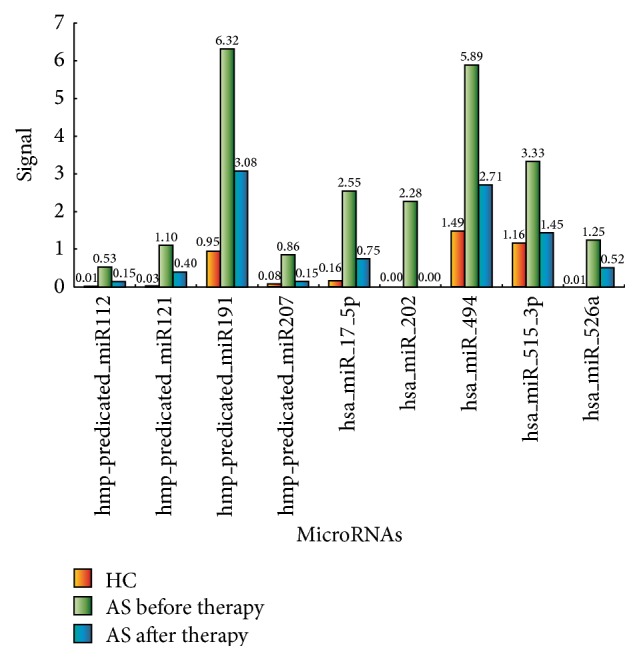
Nine microRNAs had higher expressed levels in AS group than healthy control group, and the expression levels of them downregulated after regular etanercept therapy for 12 weeks. The calibrated signal ratio of AS group before therapy to healthy control group were >3 : 1, and the ratio of AS group after therapy to before therapy were <1 : 3. HC, healthy control; AS, ankylosing spondylitis.

**Figure 3:**
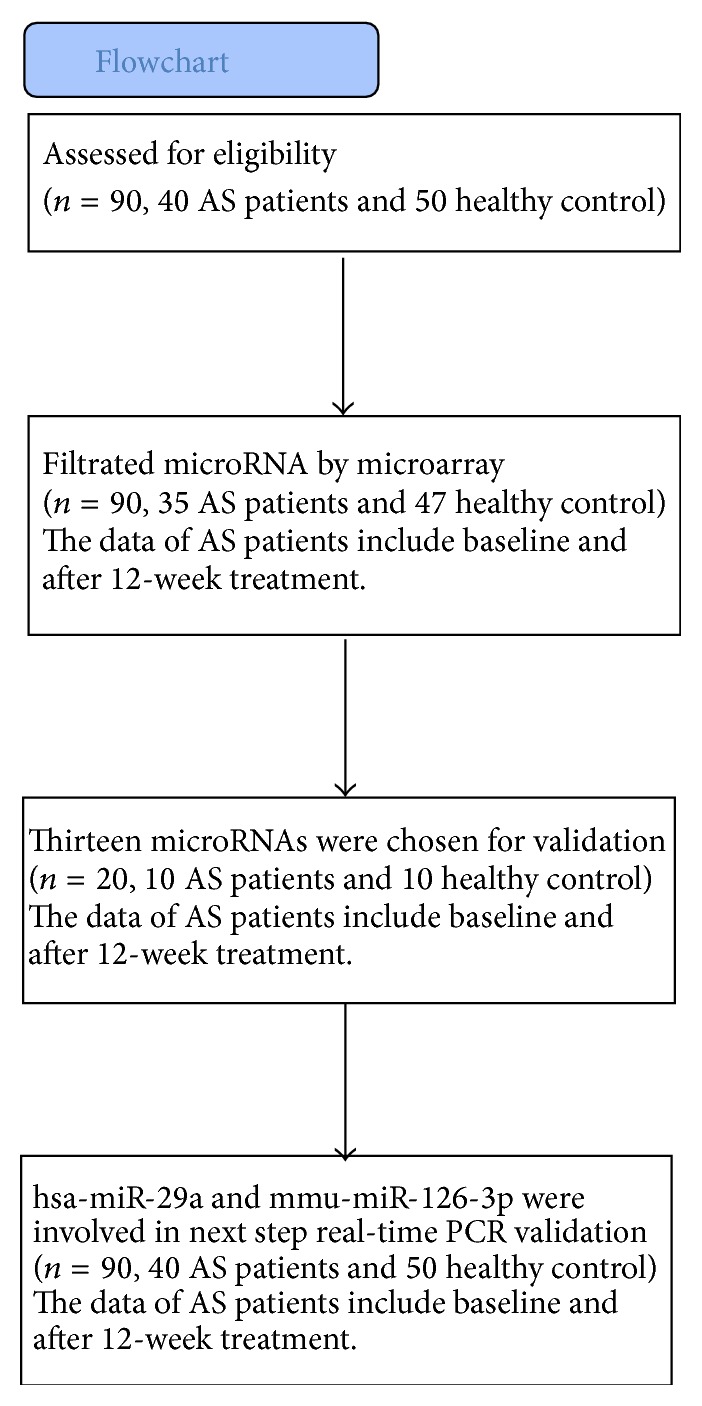


It was necessary to point out that there were 9 differentially expressed microRNAs. All of them had higher expressed levels in AS group than healthy control group. However the expression levels of these microRNAs downregulated after regular etanercept therapy for 12 weeks (Figure 2).

### 3.3. The Result of Real-Time PCR

Real-time PCR was performed on 13 candidate microRNAs (*n* = 10 for both AS group and healthy control) (Table 2). The expression level of hsa-miR-126-3p, hsa-miR-29a, and hsa-miR-let7i which was validated by real-time PCR was consistent with the result of microarray. The fold change of them was >2, and *P* value was <0.05.

Further validated study involved the 2 microRNA, hsa-miR-126-3p and hsa-miR-29a. As we anticipated, the result of further study was infusive. The expression levels of these 2 microRNAs were significantly lower in AS group (PCR values: 3.52 ± 3.76 and 7.26 ± 5.18; *n* = 40 and 29 for hsa-miR-126-3p and hsa-miR-29a, resp.) than in control group (PCR values: 5.42 ± 3.71 and 11.28 ± 0.62, *n* = 50 and 30 for hsa-miR-126-3p and hsa-miR-29a, resp.). Marched fold changes of them were 3.76 and 16.22. Moreover, expressions of hsa-miR-126-3p and hsa-miR-29a were dramatically upregulated after 12-week etanercept treatment. Fold changes were 2.20 and 3.18 (*n* = 30 and 29 for hsa-miR-126-3p and hsa-miR-29a, resp.). All the regulations of microRNAs expression mentioned before were statistically significant (fold change >2, *P* < 0.05) (Table 3).

### 3.4. Correlation Analysis of MicroRNAs and Clinical Presentation

Correlation analysis of 2 microRNAs, hsa-miR-126-3p and hsa-miR-29a, expression and clinical data indicated that hsa-miR-126-3p expression disorders statistically significantly correlated with CRP and ESR (Table 4).

## 4. Discussion

Since the discovery of microRNA, lin-4, in 1993 at Harvard [15], a great deal of effort had been devoted to annotate their biologic function and the relevance to diseases. Not only had the genomics, biogenesis, mechanism, and function of microRNAs been discovered [16], but also disorders of microRNAs had been associated with certain human disease and pathological changes of tissue. MicroRNAs thought to take part in pathologic process of 15 common human disorders [12]. Recent studies suggested that miRNAs in PBMCs could be biomarkers for the diagnosis of heart disease [17] and prostate cancer [18, 19]. MicroRNAs were also suggested to be potential biomarkers for drug-induced liver injury [20] and myocardial injury [21]. Furthermore, microRNAs were proved to play an important role in pathogenesis, diagnosis, and therapy of rheumatism. For examples, synovial fluid and plasma microRNAs had potential as diagnostic biomarkers for RA and OA and as a tool for the analysis of their pathogenesis [10], while miR-146a in lupus patients was relevant to the biologic and clinical behavior of SLE. And microRNA could serve as therapeutic targets for the treatment of SLE via regulation of the type I IFN pathway [11]. An article mentioned that miR-125 and another 6 microRNAs may have correlation with pathological changes of AS [12]. But no further confirmatory experiment had been done. In this report, we showed the PBMCs microRNAs expressions profiles of AS patients were distinct from these of healthy donors. And after regular etanercept therapy, the dysregulation of microRNAs expression could be corrected. Finally, we discussed the possibility of PBMCs miRNAs to be potential biomarkers of AS.

Our hypothesis was that microRNAs took part in pathogenesis of AS. According to this hypothesis, we could infer that (a) expression of microRNAs was disorder in active stage of AS; (b) when the clinical presentations were controlled, the expression of microRNAs would tend to be normal. For one thing, the expression levels of hsa-miR-126-3p and hsa-miR-29a were lower in AS group than in healthy control group. The differences between the two groups were statistically significant. And the expressions of hsa-miR-126-3p and hsa-miR-29a were significantly upregulated in AS group after 12-week therapy taking baseline as control. Unfortunately, it was not certified that expression disorders of microRNAs and clinical parameters of AS activity (BASDAI, CRP, and ESR) correlated linearly. At least there were three reasons that shouldered the responsibility. Firstly, microRNAs disorders and alterations of clinical parameters maybe have complex correlation, not linear, which would be affected and regulated by many factors. Secondly, clinical parameters reflected just inflammatory activity of AS, while microRNAs disorders may be related to bone metabolism. Relative clinical parameters could be collected to validate this hypothesis in the future. Thirdly, appearance of microRNAs disorders and clinical presentations may lack temporal concurrence, while data collection was performed at the same time point. Under the circumstances the result would not reflect their real correlation.

On the basis of our hypothesis, microRNAs, hsa-miR-126-3p and hsa-miR-29a, are probably potential diagnostic biomarkers for AS. As reported in the field of malignant tumors [18, 22, 23], disease specific miRNAs for AS are expected. In our study, we proved that expression levels of microRNAs could differentiate AS from healthy control. When the clinical presentations were controlled, the expression of microRNAs would tend to be normal. The variation trend of microRNAs and course of disease were of high degree of consensus. Quantitative analysis of microRNAs mentioned before would be of great value in AS diagnosis, activity evaluating, and curative effect monitoring. Furthermore, regular etanercept therapy could elevate the downregulated microRNAs of AS patient, and this change was also statistically significant. It is suggested that hsa-miR-126-3p and hsa-miR-29a have potential to be new target for AS treatment.

Our research provides clues of further researches. MicroRNAs play a special role in gene regulation [24]. MiR-29 was thought to be involved in the regulation of collagen expression in hepatic stellate cells [25] and fibroblasts [26], though the target site was not revealed. MiR-126 was reported to target TOM1, which was a negative regulator of IL-1b and TNF-a-induced signaling pathways [27]. There was little known information about the effect of these microRNAs on AS pathogenesis. The functions of these dysregulated microRNAs would be the emphases of sequential studies. We will focus on the target sites they are combining and the pathways they are making effect on. Target genes and their functions are expected to be revealed.

## 5. Conclusion

PBMCs microRNAs expressions profile of AS patients was distinct from these of healthy donors. Our study first verified AS pathogenesis involved dysregulation of microRNAs. Expression levels of two microRNAs, hsa-miR-126-3p and hsa-miR-29a, were distinct from these of healthy controls. And after regular etanercept therapy, the dysregulation of microRNAs expression could be corrected. They will probably become not only the potential biomarkers for AS diagnosis, activity evaluation, and curative effect monitoring, but also provocative therapeutic targets of AS.

## Figures and Tables

**Table 1:** Clinical features of the AS participants.

Characteristics	AS (*n* = 40)
Disease duration, mean ± SD years	7.9 ± 0.8
BASDAI, mean ± SD	5.25 ± 1.62
BASFI, mean ± SD	46.5 ± 23.6
CRP, mean ± SD mg/L	30.5 ± 23.5
ESR, mean ± SD mm	39.9 ± 29.5
Medications before etanercept therapy, taking/not taking	
Steroids, last 3 months	6/34
DMARDs, last 3 months	27/13
NSAIDs, last 1 month	29/11

AS, ankylosing spondylitis; HC, healthy control; SD, standard deviation; BASDAI, Bath Ankylosing Spondylitis Disease Activity Index; BASFI, Bath Ankylosing Spondylitis Functional Index; CRP, C-reactive protein; ESR, erythrocyte sedimentation rate; DMARDs, disease-modifying antirheumatic drugs; NSAIDs, nonsteroidal anti-inflammatory drugs.

**Table 2:** Expression level of microRNAs in both AS and control group validated by real-time PCR.

microRNA	2^ΔΔ*C*^_*T*_	
	AS group/control group (interval)	AS group before/after therapy (interval)
hsa-miR-202	1.28 (0.28~5.88)	0.50 (0.13~2.08)
hsa-miR-21	0.51 (0.11~2.5)	—
hsa-miR-26b^**^	0.70 (0.26~1.89)	3.43 (1.20~9.85)
hsa-miR-27a^*^	0.07 (0.01~0.58)	0.8 (0.27~2.43)
hsa-miR-29a^**^	0.26 (0.12~0.53)	0.35 (0.25~1.24)
hsa-miR-29b^**^	1.22 (0.67~2.22)	0.43 (0.19~0.97)
hsa-miR-494^**^	0.58 (0.52–5.6)	0.21 (0.05~0.89)
hsa-miR-526a^**^	1.20 (0.48~3.03)	0.40 (0.13~1.14)
hsa-miR-98^*^	0.44 (0.10~1.89)	—
hsa-miR-let7a	0.91 (0.16~4.76)	—
hsa-miR-let7f	0.54 (0.11~2.70)	—
hsa-miR-let7i^∗,∗∗^	0.27 (0.05~1.49)	0.27 (0.05~1.47)
hsa-miR-126-3p^*^	0.15 (0.03~0.75)	0.51 (0.28~0.93)

^*∗*^MicroRNAs had statistically significantly different expression in AS group and healthy control group (fold changes >2, *P* < 0.05).

^**^Expression levels were changed statistically significantly after medicine treatment (fold changes >2, *P* < 0.05).

**Table 3:** Expression of two microRNAs by real-time PCR validated.

microRNA	2^ΔΔ*C*^_*T*_			
	Control group/AS group (fold)	*P* value	AS group before/after therapy (fold)	*P* value
hsa-miR-29a	16.22 (16.22)	0.002	0.31 (3.18)	0.049
hsa-miR-126-3p	3.76 (3.76)	0.046	0.45 (2.20)	0.035

The expression levels of these 2 microRNAs were significantly lower in AS group than in control group. And expressions of them were dramatically upregulated after 12-week etanercept treatment (fold changes >2, *P* < 0.05).

**Table 4:** Correlation of 2 validated microRNAs and clinical presentation.

	BASDAI		CRP		ESR	
	Correlation coefficients	*P*	Correlation coefficients	*P*	Correlation coefficients	*P*
hsa-miR-126-3p	0.302	0.059	0.317	0.046	0.378	0.016
hsa-miR-29a	−0.038	0.843	0.207	0.282	0.153	0.429

MicroRNAs expression disorders did not statistically significantly correlated with BASDAI, CRP, or ESR. BASDAI, Bath Ankylosing Spondylitis Disease Activity Index; CRP, C-reactive protein; ESR, erythrocyte sedimentation rate; correlation coefficients, Spearman correlation coefficients; *P*, *P* value.

## Abbreviation

RNA: Ribonucleic acid
PBMC: Peripheral blood mononuclear cell
AS: Ankylosing spondylitis
PCR: Polymerase chain reaction
BASDAI: Bath Ankylosing Spondylitis Disease Activity Index
BASFI: Bath Ankylosing Spondylitis Functional Index
CRP: C-reactive protein
ESR: Erythrocyte sedimentation rate
DMARDs: Disease-modifying antirheumatic drugs
NSAIDs: Nonsteroidal anti-inflammatory drugs
HLA: Human leukocyte antigen
RA: Rheumatoid arthritis
OA: Osteoarthritis
SLE: Systemic lupus erythematosus
VAS: Visual Analogue Score
EDTA: Ethylenediaminetetraacetic acid
OD: Optical density
IFN: Interferon
IL: Interleukin
TNF: Tumor necrosis factor
HC: Healthy control
SD: Standard deviation.

## Foundations

Jieruo Gu has received grant support from the National Natural Sciences Foundation of China (30872328), Clinical Subject (2007 and 2010) of Ministry of Public Health of China and from the Foundation of Guangdong province of China (2009B080701086) and 5010 Subject of Sun Yat-sen University (2009-2010).
